# *MDR1* siRNA loaded hyaluronic acid-based CD44 targeted nanoparticle systems circumvent paclitaxel resistance in ovarian cancer

**DOI:** 10.1038/srep08509

**Published:** 2015-02-17

**Authors:** Xiaoqian Yang, Arun K. lyer, Amit Singh, Edwin Choy, Francis J. Hornicek, Mansoor M. Amiji, Zhenfeng Duan

**Affiliations:** 1 Sarcoma Biology Laboratory, Center for Sarcoma and Connective Tissue Oncology, Massachusetts General Hospital and Harvard Medical School, Boston, MA 02114; 2 Department of Gynaecology and Obstetrics, The Third Affiliated Hospital of Zhengzhou University, Zhengzhou 450052, Henan Province, China; 3 Department of Pharmaceutical Sciences, School of Pharmacy, Northeastern University, Boston, MA 02114

## Abstract

Development of multidrug resistance (MDR) is an almost universal phenomenon in patients with ovarian cancer, and this severely limits the ultimate success of chemotherapy in the clinic. Overexpression of the *MDR1* gene and corresponding P-glycoprotein (Pgp) is one of the best known MDR mechanisms. *MDR1* siRNA based strategies were proposed to circumvent MDR, however, systemic, safe, and effective targeted delivery is still a major challenge. Cluster of differentiation 44 (CD44) targeted hyaluronic acid (HA) based nanoparticle has been shown to successfully deliver chemotherapy agents or siRNAs into tumor cells. The goal of this study is to evaluate the ability of HA-PEI/HA-PEG to deliver *MDR1* siRNA and the efficacy of the combination of HA-PEI/HA-PEG/*MDR1* siRNA with paclitaxel to suppress growth of ovarian cancer. We observed that HA-PEI/HA-PEG nanoparticles can efficiently deliver *MDR1* siRNA into MDR ovarian cancer cells, resulting in down-regulation of *MDR1* and Pgp expression. Administration of HA-PEI/HA-PEG/*MDR1* siRNA nanoparticles followed by paclitaxel treatment induced a significant inhibitory effect on the tumor growth, decreased Pgp expression and increased apoptosis in MDR ovarian cancer mice model. Our findings suggest that CD44 targeted HA-PEI/HA-PEG/*MDR1* siRNA nanoparticles can serve as a therapeutic tool with great potentials to circumvent MDR in ovarian cancer.

Ovarian cancer is a lethal gynecological malignancy and ranks as the fifth leading cause of cancer death among women. It is estimated that about 21,980 women will be diagnosed with ovarian cancer and 14,270 patients will die in the United States in 2014^1^,^2^. Since most early stage ovarian cancer is generally asymptomatic, it is difficult to recognize this disease until it is at an advanced stage. Approximately 75 percent of women diagnosed with ovarian cancer will present with advanced disease. The overall five-year survival rate of patients diagnosed at stage III and IV of this disease is 32% and 18%^3^,^4^. Recommended management of advanced disease usually consists of primary cytoreductive surgery followed by paclitaxel-platinum combination chemotherapy. Although the response rate to paclitaxel and platinum is up to 80% and a great improvement of median overall survival has been made in patients treated with chemotherapy, unfortunately, 50–75% of patients with advanced ovarian cancer will ultimately relapse due to the development of multidrug resistance (MDR)^4^,^5^,^6^. MDR remains one of the most significant challenges in our endeavor to cure ovarian cancer^4^,^7^,^8^,^9^.

MDR is a phenomenon in which, after exposure of the tumor to a specific chemotherapy drug, tumor cells eventually develop resistance to that chemotherapy or to a wide range of functionally and structurally unrelated chemotherapeutic agents^8^,^10^. MDR can be either intrinsic or acquired through exposure over time to anticancer drugs, and the mechanisms of MDR are complicated^7^,^11^. Overexpression of MDR gene 1 (*MDR1*) and the corresponding ATP-binding cassette (ABC) transporter P-glycoprotein (Pgp) is one of the best characterized ways MDR can develop^8^,^10^,^12^. Pgp is an energy-dependent drug efflux pump, whose function is to export chemotherapy drugs from the inside of cells to the outside. Overexpression of Pgp will result in a decrease of intracellular accumulation of chemotherapeutic drugs, and subsequently induce MDR in tumor cells to anticancer drugs^8^,^13^. Increased expression levels of Pgp or its RNA transcript have been found in ovarian cancer, especially in MDR ovarian cancer^8^,^14^,^15^. It has been reported that *MDR1*/Pgp may have useful predictive value in determining the clinical outcome of patients with advanced ovarian cancer^16^,^17^,^18^. The expression level of *MDR1* in patients that do not respond to chemotherapy was significantly higher than in patients that respond to chemotherapy^18^. The overall survival time was statically shortened in patients with tumors with high *MDR1*/Pgp expression levels^18^. New experimental evidence has demonstrated that the epithelial-mesenchymal transition (EMT) has the potential to confer stem-cell like properties to a subpopulation of cancer cells, leading to the onset of resistance to anticancer drugs^19^,^20^. In addition, studies have also suggested that evasion of apoptosis, activation of DNA repair, activation of detoxifying system, and changes in drug transport may contribute to the development of MDR^20^.

In order to improve the outcome of chemotherapy, strategies to circumvent MDR have been studied extensively in recent years. One of the promising approaches to overcoming MDR is by inhibiting *MDR1* expression by using RNA interference (RNAi) technologies with either small interfere RNA (siRNA) or small hairpin RNA (shRNA). We and others previously have demonstrated that knocking down *MDR1* expression by transfecting MDR cells with *MDR1* siRNA using Lipofectamine or nanoparticles can restore sensitivity to paclitaxel and other Pgp substrates^21^,^22^,^23^,^24^. However, targeted, systemic, effective, and safe delivery of siRNA to a specific tumor site *in vivo* is still a major obstacle in developing siRNA as a clinically viable method to treat cancer patients with MDR.

Hyaluronic acid (HA) is an anionic, non-sulfated glycosaminoglycan. HA plays an important role in cell growth, proliferation and adhesion^25^,^26^. Cluster of differentiation 44 (CD44), a family of multifunctional trans-membrane glycoproteins, has been demonstrated to interact with HA at the N terminus of its extracellular domain, and therefore serves as a major cell surface receptor for HA^25^. The binding of HA to CD44 is important for its biological activities, including cell adhesion, migration, invasion, proliferation, and angiogenesis^25^,^26^. The presence of high expression levels of CD44 was considered to be associated with drug resistance during ovarian cancer metastasis and is an unfavorable prognosis or survival marker in ovarian cancer^26^,^27^. Co-overexpression of CD44 and Pgp has been associated with drug resistance and progression in different tumor cells including ovarian cancer^28^,^29^,^30^.

Previously, HA-based chemotherapy drugs have shown antitumor activity *in vivo*^31^. CD44 targeted HA-based nanoparticles have also been shown to successfully deliver siRNA into tumor cells and result in specific mRNA knockdown in lung cancer xenograft models^32^,^33^. In the current study, we studied the efficiency of siRNA delivery of *MDR1* siRNA loaded HA-PEI/HA-PEG CD44 targeted nanoparticles and their effects on circumventing drug resistance in an MDR ovarian cancer model *in vitro* and *in vivo*. This is the first study to examine the potential for CD44 targeted HA-PEI/HA-PEG-based nanoparticle to deliver *MDR1* siRNA to tumor cells *in vivo* to overcome drug resistance. As *MDR1* is the major mechanism for paclitaxel resistance, the combination of *MDR1* siRNA CD44 targeted nanoparticle and paclitaxel hold great potential to increase paclitaxel sensitivity/efficacy and overcome MDR in ovarian cancer treatment.

## Results

### Ovarian cancer cell lines and tumor tissues expressed high levels of CD44

**Figure 1:**
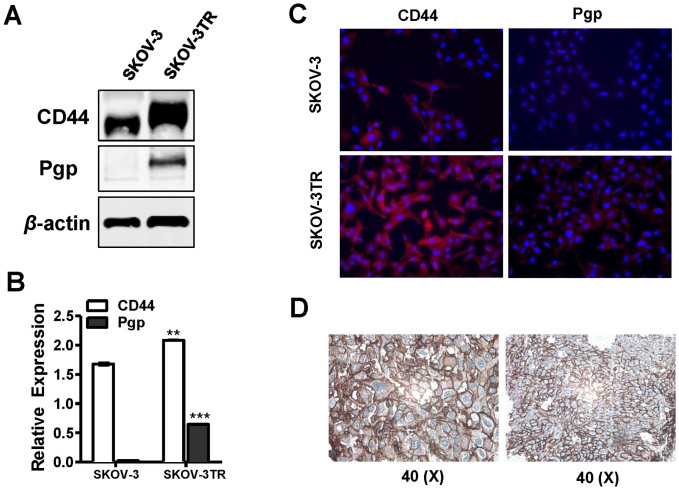
Expression of CD44 and Pgp in ovarian cancer cells and tumor tissues. (A). Expression level of CD44 and Pgp in ovarian cancer resistant cell line SKOV-3TR and parental sensitive cell line SKOV-3 determined by Western Blot. (B). Relative expression of CD44 and Pgp from (A) were analyzed by densitometry. Data are presented as means ± SEM and analyzed using Student's t-test. Statistical comparisons between SKOV-3TR and SKOV-3 cells are displayed: ** *P* < 0.01,*** *P* < 0.005. (C). Expression of CD44 and Pgp in SKOV-3TR and SKOV-3 cells evaluated by immunofluoresecence. CD44 and Pgp proteins were stained using secondary antibody conjugated with Alexa Fluor 594 (Red). Nuclei were stained with DAPI nuclear dye (Blue). (D). Expression of CD44 in ovarian cancer tissues. Representative images of IHC results were shown.

HA has high specificity and affinity for the CD44 receptor which is overexpressed on the cell surface of numerous tumors^25^. CD44 enhances the ability of HA based nanoparticles to deliver siRNA and/or drugs to the specific tumor sites^33^. We first determined the expression level of CD44 in ovarian cancer drug sensitive and resistant cell lines SKOV-3 and SKOV-3TR. We showed that both SKOV-3 parental sensitive cell line and chemoresistant cell line SKOV-3TR displayed high expression of CD44, while SKOV-3TR cells showed higher expression level of CD44 than SKOV-3 cells (Fig. 1A). Relative expression of CD44 was analyzed by densitometry and shown in Fig. 1B. However, only chemoresistant cells SKOV-3TR showed overexpression of Pgp which is consistent with previous studies that show that overexpression of Pgp is one of the major mechanisms of MDR in SKOV-3TR cell line (Fig. 1A and B). Immunofluorescence studies further confirmed that CD44 could be detected in both sensitive and resistant cells (Fig. 1C) and in ovarian cancer tissues (Fig. 1D). In addition, the expression levels of CD44 and Pgp were examined in various ovarian cancer cell lines, including drug sensitive and resistant ovarian cancer cell lines, and the results were shown in the Fig. S1.

### Characterization of *MDR1* siRNA loaded HA-PEI/HA-PEG nanoparticles

**Figure 2:**
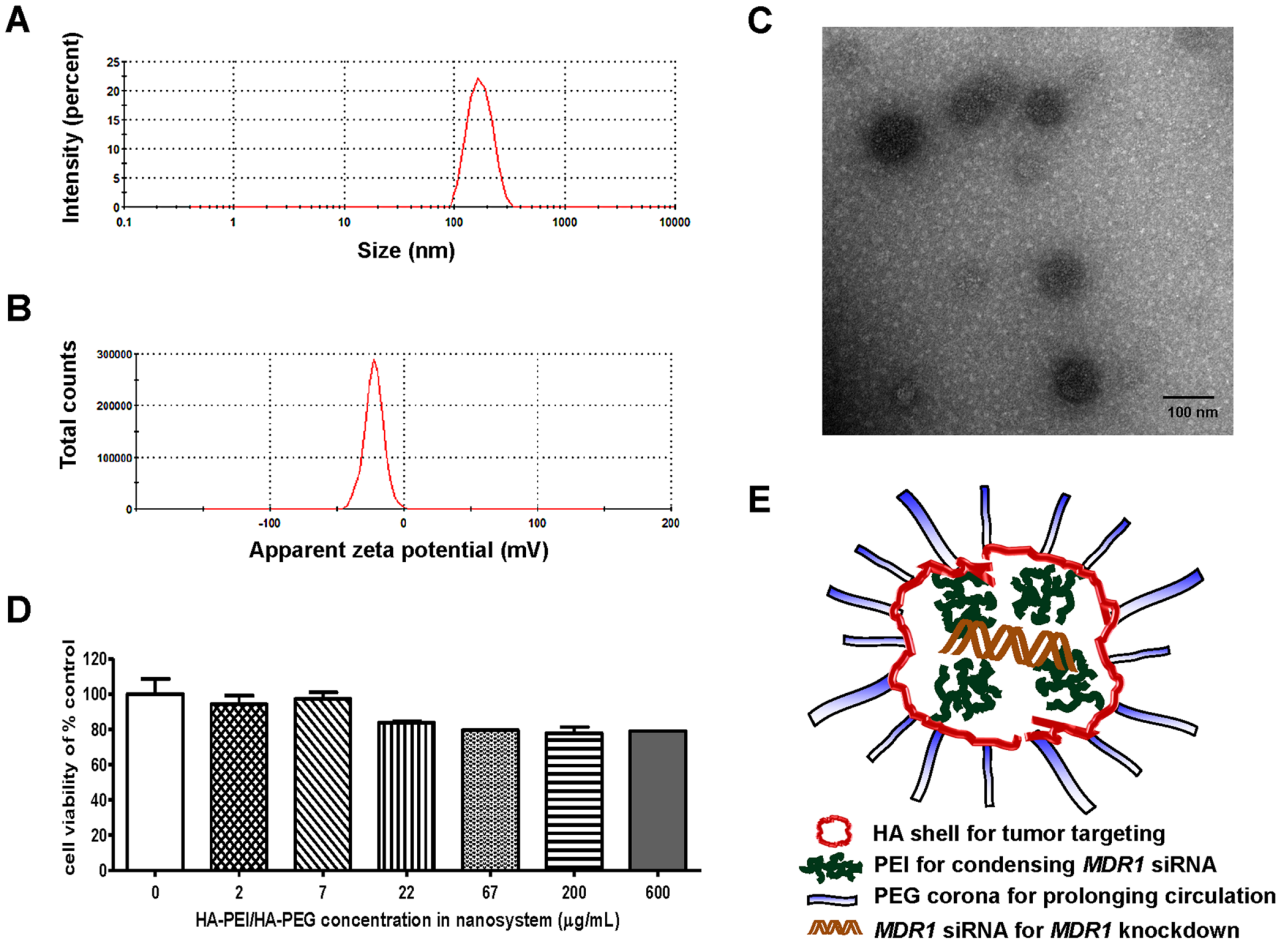
Characterization of *MDR1* siRNA loaded HA-PEI/HA-PEG nanoparticles. (A). The mean of Z-average particle size of HA-PEI/HA-PEG/*MDR1* siRNA nanoparticles was 173.3 ± 13.7 nm. (B). The zeta potential of nanoparticles was −22.5 ± 0.44 mV. (C). Representative TEM image of HA-PEI/HA-PEG/*MDR1* siRNA nanoparticles was shown. The dark core was exhibited in the central of nanoparticles. (D). The cytotoxicity of HA-PEI/HA-PEG/*MDR1* siRNA nanoparticles in SKOV-3TR cells. (E). Schematic illustration of HA-PEI/HA-PEG *MDR1* siRNA nanoparticle showed that the structure of nanoparticle was core–shell–corona. The PEG corona surrounded HA shell, and positive core contained PEI as well as *MDR1* siRNA.

The particle size and zeta potentials of *MDR1* siRNA loaded HA-PEI/HA-PEG self-assemble nanoparticles were examined using the Zetasizer Nano-S® instrument. The mean Z-average particle size (nm) of HA-PEI/HA-PEG/*MDR1* siRNA nanoparticles was 173.3 ± 13.7 nm (Fig. 2A). As shown in Fig. 2B, a negative surface charge was presented by HA-PEI/HA-PEG/*MDR1* siRNA nanoparticles. The zeta potential of nanoparticles was −22.5 ± 0.44 mV, despite the presence of cationic polymer, PEI. Representative TEM image suggested a dark core for the nanoparticles, which may be attributed to the high contrast arising from the uranyl acetate stained *MDR1* siRNA loaded in the core of the HA-PEI/HA-PEG nanoparticles (Fig. 2C).

One of the key questions concerning nanocarriers is the safety for future clinical application. Therefore, the cytotoxicity of HA-EPI/HA-PEG/*MDR1* siRNA for SKOV-3TR cells was evaluated by MTT assay prior to gene silencing tests. SKOV-3TR cells were plated in 96-well plate and incubated with HA-PEI/HA-PEG/*MDR1* siRNA for 6 h on the following day. The cells were subsequently incubated with fresh media for another 48 h and MTT assay was performed. The results showed 80% of the cells remained viable after treatment with HA-PEI/HA-PEG/*MDR1* siRNA for 6 h at the concentration of 600 µg/mL, while in current study, the highest concentration of HA-PEI/HA-PEG used for transfection was 170 µg/mL. This finding suggested that there was no apparent cytotoxicity of HA-PEI/HA-PEG/*MDR1* siRNA during transfection and the cells could tolerate it well (Fig. 2D). Based on the results as described above, schematic illustration of HA-PEI/HA-PEG/*MDR1* siRNA nanoparticle was shown in Fig. 2E. The structure of nanoparticle has a core–shell architecture with a PEG corona. The PEG corona surrounds the hydrophilic HA shell with the core containing the positively charged PEI complexed with the negatively charged *MDR1* siRNA.

### Suppression efficacy of target gene using *MDR1* siRNA loaded HA-PEI/HA-PEG CD44 targeted nanoparticles

**Figure 3:**
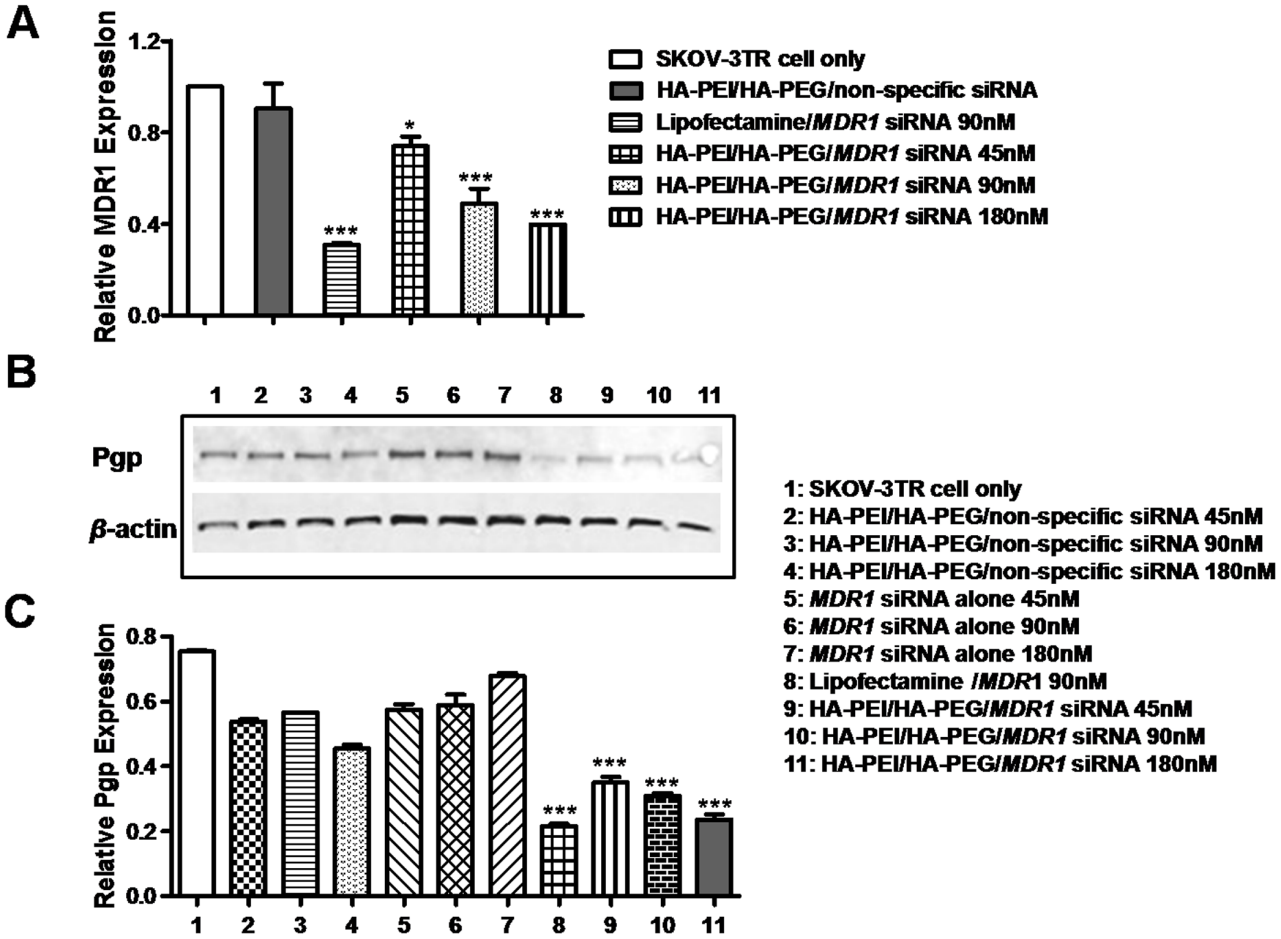
Expression level of target gene in SKOV-3TR cells transfected with HA-PEI/HA-PEG/*MDR1* siRNA nanoparticles. (A). Expression level of *MDR1* mRNA in HA/PEI/HA-PEG/*MDR1* siRNA transfected cells. *MDR1* mRNA expression level was determined by real-time PCR and the data was analyzed using 2^−ΔΔCt^ method. (B). Influence of HA-PEI/HA-PEG/*MDR1* siRNA on Pgp expression. SKOV-3TR cells were incubated with different agents indicated. (C). Densitometric quantification data from Western blot results. Means ± SEM were shown. Data are presented as means ± SEM and analyzed using Student's t-test. * *P* < 0.05, *** *P* < 0.005.

In order to ascertain if the *MDR1* siRNA was delivered and released from HA-PEI/HA-PEG CD44 targeted nanoparticle and still maintained its functional activity to knockdown gene expression *in vitro*, the RNA was extracted from SKOV-3TR cells after transfected with HA-PEI/HA-PEG/*MDR1* siRNA. Real-time PCR assay was conducted and the expression level of *MDR1* targeted gene was assessed. We showed that HA-PEI/HA-PEG/*MDR1* siRNA nanoparticle transfected cells showed significantly decreased levels of *MDR1* expression as determined by relative quantitative analysis using 2^−ΔΔCt^ method. However, no significant changes of *MDR1* expression were observed in cells incubated with either *MDR1* siRNA alone or HA-PEI/HA-PEG CD44 targeted non-specific siRNA. In addition, the level of decrease in *MDR1* expression was correlated with the dosage of *MDR1* siRNA loaded nanoparticle in a dose dependent manner. Indeed, the expression levels of *MDR1* in cells transfected with 45 nM, 90 nM and 180 nM *MDR1* siRNA decreased by 73.9%, 48.5% and 39.6%, respectively, when compared with the SKOV-3TR cells without treatment (Fig. 3A). Similar results were found in OVCAR8TR cells (another MDR ovarian cancer cell line, data not shown).

The human *MDR1* gene encodes an integral membrane protein, Pgp. Therefore, expression levels of Pgp in cells transfected with CD44 targeted HA-PEI/HA-PEG/*MDR1* siRNA were also evaluated. The expression level of Pgp significantly decreased in SKOV-3TR cells transfected with HA-PEI/HA-PEG/*MDR1* siRNA nanoparticles. Western blot analysis revealed that this inhibition was also dose-dependent (Fig. 3B). Further densitometric quantification data from Western blot results are shown in Fig. 3C. The expression level of Pgp in cells transfected with 45 nM, 90 nM and 180 nM MDR1 siRNA decreased by 46.4%, 41.1% and 31.4%, respectively, when compared with that the cells without transfection. Similar results were found in OVCAR8TR cells (Fig. S2). Taken together, these data, demonstrated that *MDR1* siRNA was not only delivered and released from HA-PEI/HA-PEG CD44 targeted nanoparticle but also retained its activity to efficiently silence *MDR1* gene and Pgp expression.

### Combination of HA-PEI/HA-PEG/*MDR1* siRNA CD44 targeted nanoparticle and paclitaxel treatment inhibited resistant ovarian cancer tumors growth

**Figure 4:**
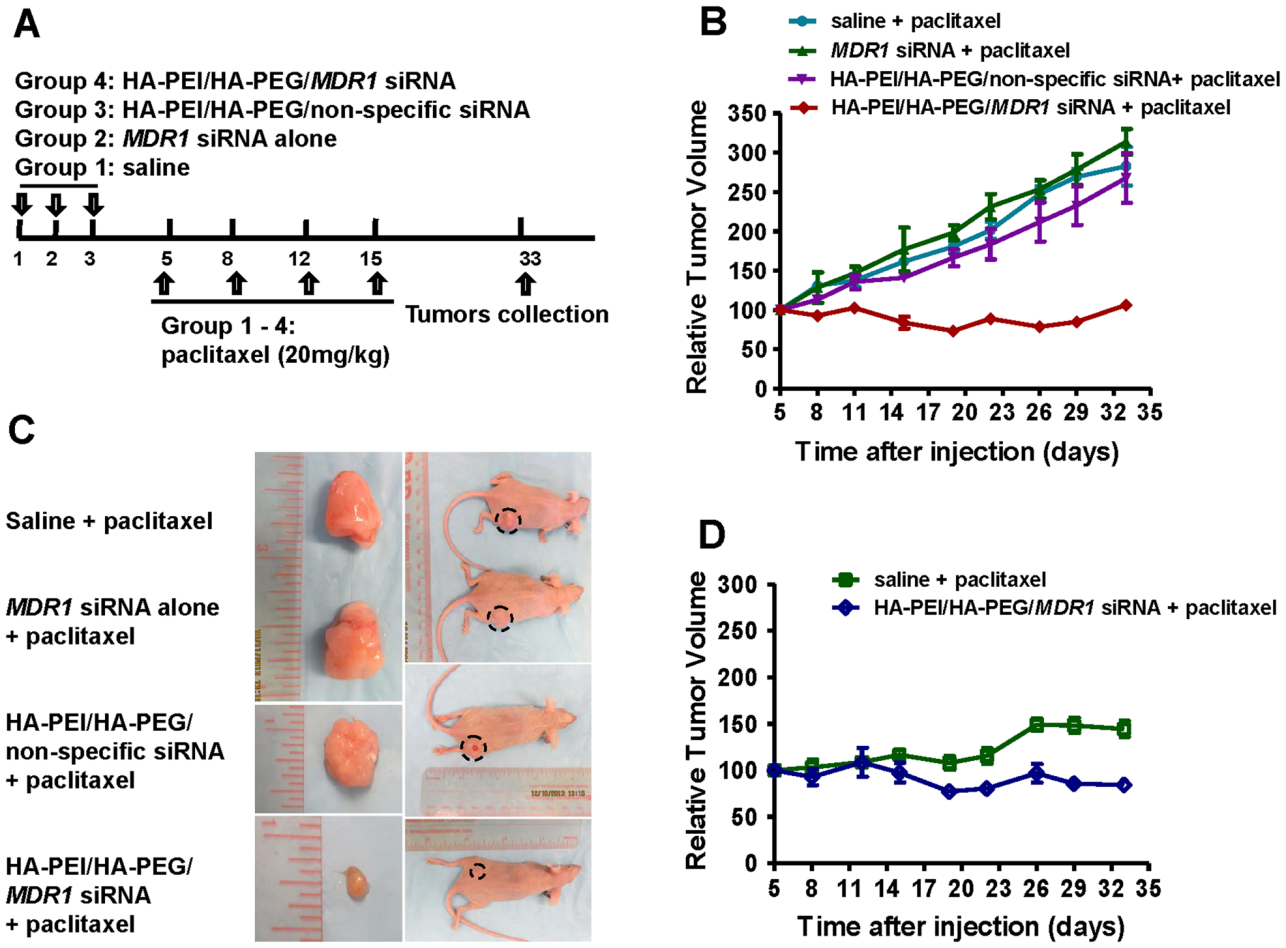
*In vivo* efficacy of the co-administered HA-PEI/HA-PEG/*MDR1* siRNA and paclitaxel treatment on resistant tumor growth. (A). Treatment scheme of SKOV-3TR model was listed. The day that treatment was initiated was defined as day 1. The mice were treated with either saline, *MDR1* siRNA, non-specific siRNA encapsulated in HA-PEI/HA-PEG or *MDR1* siRNA encapsulated in HA-PEI/HA-PEG at a dose of 0.5 mg/kg on day 1, day 2 and day 3. On day 5, all groups were followed with paclitaxel (20 mg/kg) treatment twice a week for two weeks. The tumors were collected on day 33. (B). The tumor growth curve of mice bearing xenografts of human ovarian cancer multidrug resistant cells SKOV-3TR. Mice were administrated with drugs indicated. The tumors volume (V_t_, mm^3^) was measured regularly twice a week and calculated using the formula (W^2^ × L)/2, where W represented width and L represented length. The relative tumor volume was calculated using the formula V_t_/V_0_, where V_0_ was tumor volume at time of treatment starting. (C). Representative tumors from each group were listed. (D). Mice bearing xenografts of human ovarian cancer cells SKOV-3 cells were treated with either HA-PEI/HA-PEG/non-specific siRNA or HA-PEI/HA-PEG/*MDR1* siRNA nanoparticles followed with paclitaxel. HA-PEI/HA-PEG/*MDR1* siRNA followed with paclitaxel treatment had no obvious effects on tumor growth in SKOV-3 xenograft model.

After confirming that HA-PEI/HA-PEG CD44 targeted nanoparticles can efficiently deliver MDR1 siRNA into MDR cells and result in down-regulation of *MDR1* and Pgp expression, we next evaluated antitumor efficacy of the combination of HA-PEI/HA-PEG/*MDR1* siRNA CD44 targeted nanoparticles and paclitaxel in ovarian cancer tumor-bearing nude mice. The treatment scheme is shown in Fig. 4A. Our *in vivo* study showed that the average tumor volume in mice administrated with the combination of HA-PEI/HA-PEG/*MDR1* siRNA CD44 targeted nanoparticle and paclitaxel was significantly smaller than that observed in control groups (Fig. 4B). Tumor volume of mice treated with paclitaxel alone was approximately 3-fold higher than in mice treated with combined HA-PEI/HA-PEG/*MDR1* siRNA CD44 targeted nanopartricle and paclitaxel, which could reflect an increased chemosensitivity to paclitaxel in mice treated with HA-PEI/HA-PEG/*MDR1* siRNA. However, changes in tumor volume in mice treated with either the combination of HA-PEI/HA-PEG/non-specific siRNA CD44 targeted nanoparticle and paclitaxel or the combined naked *MDR1* siRNA and paclitaxel was similar to that with paclitaxel treated alone in this ovarian cancer MDR model (Fig. 4B). Representative images of mice and tumors form each group were shown in Fig. 4C.

In comparison, we also tested the effects of the combination of HA-PEI/HA-PEG/*MDR1* siRNA CD44 targeted nanoparticle and paclitaxel on tumor growth in drug sensitive SKOV-3 (not express *MDR1* and Pgp) xenograft mice model that did not highly express Pgp. We demonstrated that administration of HA-PEI/HA-PEG/*MDR1* siRNA CD44 targeted nanoparticle followed by paclitaxel treatment had similar effects on tumor growth as the control group of tumor treated with saline and paclitaxel in SKOV-3 xenograft model (Fig. 4D). These data suggest that increased chemosensitivity in combination of HA-PEI/HA-PEG *MDR1* siRNA CD44 targeted nanoparticles and paclitaxel treatment in SKOV-3TR is the result of down-regulation of *MDR1* and Pgp expression, and reversal of paclitaxel resistance. Additionally, on the basis of animal body weight and mortality, no significant toxicity was noticed and the animals appeared to have tolerated HA-PEI/HA-PEG nanoparticles well when HA-PEI/HA-PEG/*MDR1* siRNA and HA-PEI/HA-PEG/non-specific siRNA were administered (data not shown).

### Combined HA-PEI/HA-PEG/*MDR1* siRNA CD44 targeted nanoparticle and paclitaxel treatment decreased Pgp expression and increased apoptosis in MDR cells

**Figure 5:**
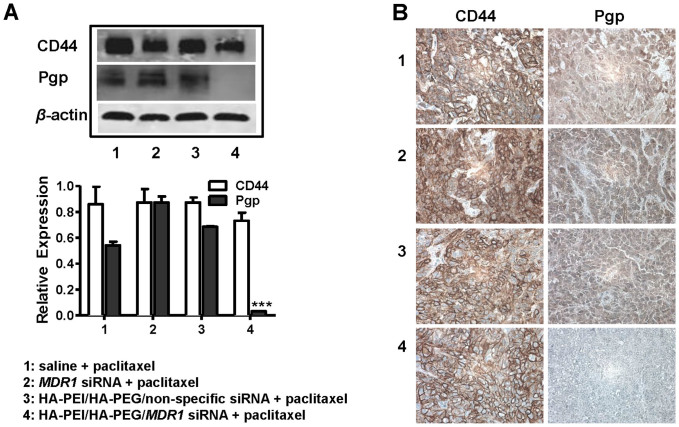
Expression level of Pgp and CD44 in SKOV-3TR mice model. (A) Representative Western blot results were presented. Administration the combination of HA-PEI/HA-PEG/*MDR1* siRNA nanoparticles and paclitaxel dramatically decreased Pgp expression in SKOV-3TR model. CD44 was showed high expression level in SKOV-3TR mice model. Densitometric quantification data from Western blot results were presented with mean ± SEM. *** *P* < 0.005. (B) Expression level of Pgp and CD44 in mice model was further evaluated by IHC. Histologic analysis of effect of HA-PEI/HA-PEG/*MDR1* siRNA on Pgp staining in ovarian cancer tumor tissues further showed downregulation of Pgp compared with either saline alone, *MDR1* alone or HA-PEI/HA-PEG/non-specific siRNA. Representative images were shown.

In order to determine the underlying mechanism of how chemosensitivity to paclitaxel increased in the xenograft MDR ovarian cancer model in mice, the proteins of SKOV-3TR tumor mass were extracted to further determine the expression levels of Pgp and CD44. We showed by western blot that administration of the combination of HA-PEI/HA-PEG/*MDR1* siRNA CD44 targeted nanoparticles and paclitaxel dramatically decreased Pgp expression in MDR cells from mice. These *in vivo* data are consistent with the findings observed *in vitro* (Fig. 5A). Immunohistochemistry analysis of HA-PEI/HA-PEG/*MDR1* siRNA CD44 targeted nanoparticle on Pgp staining in ovarian cancer MDR tumor tissues further confirmed downregulation of Pgp as compared with either saline alone, *MDR1* alone or HA-PEI/HA-PEG/non-specific siRNA nanoparticle treatment (Fig. 5B). Additionally CD44 expressions were detected in ovarian cancer tumor tissues in xenograft model, consistent with the *in vitro* results (Fig. 5B).

**Figure 6:**
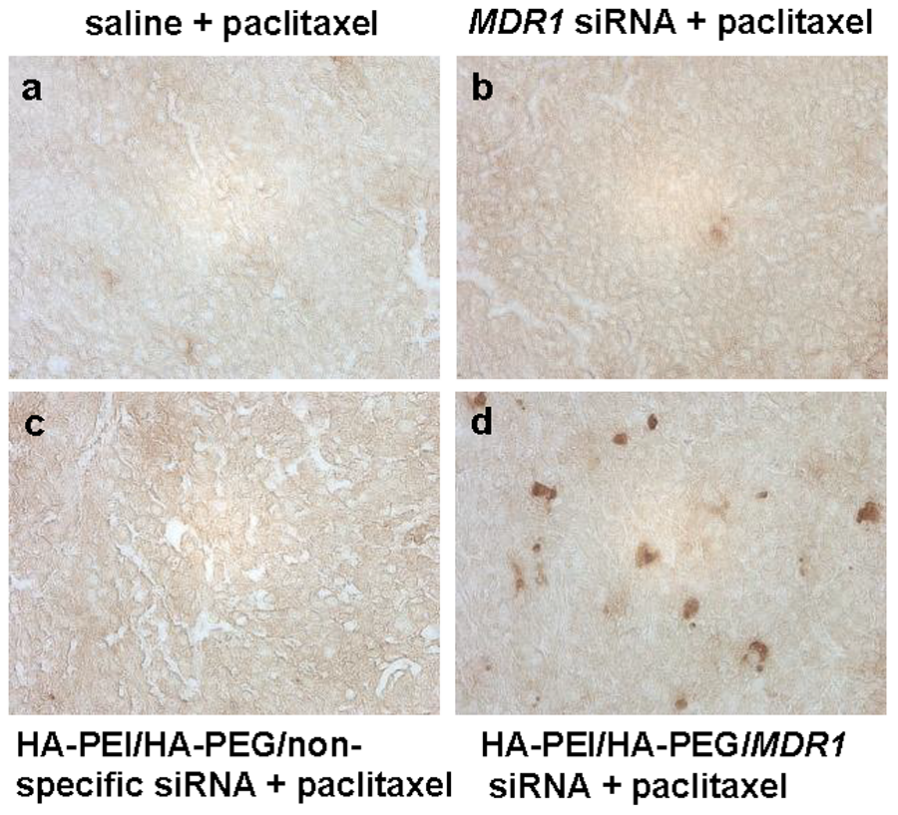
The apoptosis level in tumor tissues form mice bearing xenografts of human ovarian cancer multidrug resistant cells SKOV-3TR. Mice were administrated with either saline alone, *MDR1* siRNA alone, non-specific siRNA loaded HA-PEI/HA-PEG or *MDR1* siRNA loaded HA-PEI/HA-PEG, and followed with paclitaxel. Paraffin tissue sections of tumors were processed with the DeadEnd^TM^ Colorimetric TUNEL System. Representative images were shown.

We used TUNEL apoptosis assay on tumor sections to test whether the tumor suppression with combination HA-PEI/HA-PEG/*MDR1* siRNA CD44 targeted nanoparticle and paclitaxel treatment is related to induced apoptosis in treated tumor. As shown in Fig. 6, the apoptosis level was enhanced in the MDR ovarian tumor tissues in animals administrated with the combination of HA-PEI/HA-PEG/*MDR1* siRNA and paclitaxel. However, the apoptosis level was not significant in tumor sections for animals that received either saline alone, *MDR1* alone or HA-PEI/HA-PEG/non-specific siRNA nanoparticle followed by paclitaxel treatment.

## Discussion

Paclitaxel is one of the most effective chemotherapeutic agents for ovarian cancer and is a first line drug used in the treatment of this disease; however, the eventual development of resistance to paclitaxel therapy occurs almost in all patients^8^,^9^,^12^. Overcoming Pgp-based MDR is one possible way to achieving greater effect from chemotherapy. Although a wide range of compounds that interact with Pgp and block drug efflux have been reported to reverse the MDR phenotype^10^,^34^, these compounds are nonspecific, toxic and have low potency at doses patients can tolerate^34^. For the majority of these compounds, clinical toxicities associated with their use at concentrations required to inhibit Pgp have precluded their widespread use for overcome MDR^10^,^35^. The results from many clinical trials showed almost no survival benefits by using these MDR reversing agents of Pgp inhibitors such as verapamil, cyclosporine-A, PSC-833 (Valspodar) and VX-710 (Biricodar) in different cancer types including ovarian cancer, highlighting a crucial need for an alternative specific, safe and effective strategy to overcome MDR^36^,^37^,^38^,^39^,^40^. In this regard, the utility of nanotechnology may lead to innovative methods for effective and targeted delivery of siRNA-based therapies to circumvent MDR.

Our results indicate that HA-PEI/HA-PEG CD44 targeted nanoparticles can efficiently deliver *MDR1* siRNA into MDR ovarian cancer cells both *in vitro* and *in vivo*. We demonstrated that HA-PEI/HA-PEG CD44 targeted nanoparticles loaded with *MDR1* siRNA efficiently down-regulate the expression of *MDR1* and Pgp, inhibit the functional activity of Pgp, and subsequently increase MDR cell sensitivity to paclitaxel. Administration of HA-PEI/HA-PEG/*MDR1* siRNA nanoparticle followed by paclitaxel treatment induced a significant inhibitory effect on the tumor growth in MDR ovarian cancer xenograft mouse model as compared with control groups. As albumin-bound paclitaxel nanoparticles (Abraxane) have been approved by the FDA for the treatment of metastatic breast cancer and non-small cell lung cancer (NSCLC)^41^,^42^, and the limited supply of the first FDA-approved nano-drug Doxil in recent years^43^, the results from our current study signifies that CD44-targeted HA-PEI/HA-PEG nanoparticle platform can be an effective delivery system for siRNA-based anticancer therapeutics in treating drug resistant ovarian cancer.

Among various nanoparticles used for siRNA delivery for systemic administration, HA-PEI/HA-PEG nanoparticle possesses unique properties. HA is a naturally occurring polyanionic biopolymer and is one of the major components of the extracellular matrix of connective tissues and present in synovial fluid of joints and in the vitreous body^44^. HA has been extensively investigated for several biomedical applications, such as tissue engineering and drug and gene delivery systems, because of its biocompatibility, biodegradability, and readily modifiable chemical structure^44^. The present work showed HA-PEI/HA-PEG nanoparticles not only hold high efficiency of *MDR1* siRNA encapsulation and delivery into tumor cells, but also displayed no significant cytotoxicity.

Another advantage of HA-PEI/HA-PEG nanoparticle is the potential of increased cellular uptake of *MDR1* siRNA in MDR ovarian cancer cells via targeted delivery through HA-CD44 receptor interaction. CD44 is a receptor for HA and has been shown to play critical roles in ovarian cancer stem cells, metastasis and drug resistance^25^,^45^. CD44 is an acidic glycoprotein which binds HA with a particularly high affinity. The binding of HA to CD44 triggers direct cross-signaling between different signaling pathways including HER2, Src kinase and ERK, and CD44 is thought to be involved in increased motility, adhesion, and invasion of cancer cells as well as differentiation of cancer stem cell^25^,^44^,^45^. Higher level of CD44 expression has been shown in ovarian cancer compared with benign and borderline tumors^46^. Several studies have suggested that cancer patients with CD44 positive tumors have a significantly shorter disease-free survival than patients with CD44 negative tumors^46^. CD44 monoclonal antibody treatment has been shown to inhibit ovarian cancer cell motility but not invasion^47^. Anti-CD44 antibody has also been shown to decrease the number of total peritoneal ovarian cancer metastases in mice^47^. In addition, previous studies have shown that in ovarian cancer cells, paclitaxel-HA conjugate interacted with CD44, entered the cells through a receptor-mediated mechanism, and exerted a concentration-dependent inhibitory effect on tumor cell growth^48^. After intra-peritoneal (IP) administration in mice, HA bioconjugate distributed uniformly within the peritoneal cavity, was well tolerated, and not associated with local toxicity^48^. HA-based CD44 targeted nanoparticle has been used for both either chemotherapy drug or siRNA delivery^31^,^32^,^48^. Overexpression of CD44 enhances tumor cell growth, cancer stem cell differentiation, drug resistance, and metastases^25^,^30^,^49^. A more recent study showed CD44 is highly overexpressed in metastatic ovarian cancer cells than the cells isolated from primary tumors^45^. Combined treatment with a nanoscale-based drug delivery system for paclitaxel, CD44 siRNA led to the suppression of CD44 mRNA and protein expression, efficient induction of cell death, and effective tumor shrinkage^45^. In this study, we tested HA-PEI/HA-PEG CD44 targeted nanoparticle for *MDR1* siRNA in ovarian cancer MDR cells with anticipation that this delivery system will result in the lowering of the apoptotic threshold necessary for augmenting the efficacy of paclitaxel in a MDR ovarian cancer model. *MDR1* siRNA was efficiently encapsulated in HA-PEI/HA-PEG nanoparticles and transfected cells showed significant decreased MDR1 expression. The resulting delivery system was initially evaluated in human ovarian cancer MDR cell line SKOV-3TR cell cultures and shown to reverse resistance to paclitaxel. Furthermore, we evaluated for therapeutic efficacy in tumor-bearing SKOV-3TR mouse model. Aqueous solutions and HA-PEI/HA-PEG nanoparticle formulations with *MDR1* siRNA, non-specific siRNA, and paclitaxel were administered in combination via the tail vein to tumor-bearing mice. We showed that the average tumor volume in mice administrated with the combination of HA-PEI/HA-PEG/*MDR1* siRNA CD44 targeted nanoparticle and paclitaxel was significantly smaller than that observed in control groups. We then observed that combined HA-PEI/HA-PEG/*MDR1* siRNA CD44 targeted nanoparticle and paclitaxel treatment decreased Pgp expression and increased apoptosis in MDR cells in tumor tissues from tumor-bearing mice.

In the current study, human ovarian cancer cell line SKOV-3TR was used as a model of drug resistant ovarian cancer cells. There were several reasons we used SKOV-3TR cells. First, SKOV-3 is one of the best characterized ovarian cancer cell lines, and the resistant variant of SKOV-3, SKOV-3TR, is also a well known drug resistant ovarian cancer cell lines. Both of these cell lines have been widely used in studies focusing on ovarian cancer drug resistance^50^,^51^,^52^. Secondly, in the current study, *MDR1* siRNA was used to knockdown the expression of *MDR1* and corresponding Pgp. It has been demonstrated that drug resistant ovarian cancer cell line SKOV-3TR overexpresses *MDR1* and Pgp, which is one of the important foundations of using our HA-based nanoparticle system^22^,^53^,^54^. Thirdly, HA can innately recognize CD44, and therefore allow for targeted delivery of HA-based *MDR1* siRNA nanoparticles into cells. As shown in the results of the present study, SKOV-3TR cells displayed higher expression level of CD44 than the parent SKOV-3 cells. HA-PEI/HA-PEG nanoparticles also successfully delivered *MDR1* siRNA into another drug resistant ovarian cancer cell line, OVCAR8TR. Interestingly, SKOV-3/SKOV-3TR cells are p53 null cells, while OVCAR8/OVCAR8TR cells express mutated p53. However, similar effects of HA-PEI/HA-PEG/*MDR1* siRNA on reversing paclitaxel resistance were obtained in these cell lines. These findings indicate that MDR1 siRNA loaded HA-based CD44 targeted nanoparticles reverse drug resistance in a p53-independent manner. Our study is consistent with previous reports suggesting that p53 status does not affect sensitivity of human ovarian cancer cell lines to paclitaxel^55^.

In conclusion, our study demonstrated that systemic administration of HA-PEI/HA-PEG/*MDR1* siRNA CD44 targeted nanoparticle and paclitaxel represent an important advance and this strategy can be effective in enhancing the sensitivity of MDR cells to paclitaxel and to overcome drug resistance in MDR ovarian cancer.

## Methods

### Cell culture, reagents and chemicals

MDR ovarian cancer cell lines SKOV3TR, OVCAR8TR used in this study have been well-characterized previously^22^,^50^,^56^,^57^. SKOV-3TR and OVCAR8TR cells were cultured in RPMI 1640 (Life Technologies, Grand Island, NY) plus 10% fetal bovine serum and 1% penicillin/streptomycin (Life Technologies, Carlsbad, CA) at 37°C in 5% CO2-95% air atmosphere. The fluorescent labeled 3′-AlexaFluor488 (AF488)-*MDR1* siRNA (target sequences: 5′-GAGCTTAACACCCGACTTACATT-3′, green color) was obtained from QIAGEN GmbH (Germany). Two unlabeled siRNAs targeting MDR1 (Genebank Accession Number NM_00927, 5′-GACCAUAAAUGUAAGGUUU-3′ and 5′-GAGCUUAACACC CGACUUAUU-3′) were purchased separately from Sigma-Aldrich (St. Louis, MO) and Life Technologies. The non-specific siRNA and Lipofectamine® RNAiMAX were also purchased from Life Technologies Corp. The monoclonal mouse anti-human Pgp antibody was acquired from Sigma-Aldrich. The monoclonal mouse anti-human CD44 antibody was purchased form Cell Signaling Technology (Beverly, MA). Paclitaxel was obtained by the pharmacy at the Massachusetts General Hospital. HA (MWw20 kDa) was purchased from Lifecore Biomedical Co. (Chaska, MN). PEI (MW10,000 Da) was obtained from Polysciences Inc. (Warrington, PA). Mono-functional PEG amine (PEG2k-NH2, MW ¼ 2000 Da) was purchased from Creative PEG Works (Winston Salem, NC). All the fatty amines and other reagents for synthesis were purchased from Acros Organics (Thermo Fisher, Pittsburgh, PA) or Sigma Aldrich Chemical Co (Milwaukee, WI) at high purity (>99%) and used without further purification.

### Synthesis of HA-PEI and HA-PEG derivatives

HA-PEI and HA-PEG were synthesized as previous described with minor modification^32^,^33^. Briefly, HA was chemically conjugated with PEI dissolved in 5 mL of formamide by warming the reaction system up to 50°C in a glass scintillation vial. Subsequently, 1-ethyl-3-[3-dimethylaminopropyl] carbodiimide hydrochloride (EDC) was added into the reaction mixture and the reaction was then stirred overnight. The resulting solution was added to a large excess of EtOH (250 mL) to precipitate the polymer. The precipitate was centrifuged and collected and the washings were discarded. To purify the polymer, EtOH precipitation and washing was repeated thrice. The precipitate polymer was finally re-dissolved in deionized water and subjected to ultrafiltration. The concentrated and purified polymer was then lyophilized using a freeze dryer and stored at −20°C. For the preparation of HA-PEG polymer, HA was chemically modified with polyethyleneglycol amine (PEG-NH2) using EDC and n-hydroxy sulfo succinimide (sulfo-NHS). The purified product was also lyophilized and stored at −20°C.

### HA-PEI/HA-PEG/*MDR1* siRNA nanoparticles preparation and characteristics

HA-PEI and HA-PEG with same mass were mixed and incubated for 5 min at room temperature to form self-assembled nanoparticles (HA-PEI/HA-PEG). We previously optimized the formulation and demonstrated that the highest gene silence efficiency was achieved when siRNA was encapsulated in HA-PEI/HA-PEG at a mass ratio of 54:1 (polymer : siRNA), therefore, the same ratio of polymer and *MDR1* siRNA was used in the current study^32^. *MDR1* siRNA was mixed with HA-PEI/HA-PEG and then incubated for another 15 min at room temperature. The particle size and zeta potentials of the *MDR1* siRNA loaded HA-PEI/HA-PEG nanoparticles were measured with Zetasizer Nano S instrument (Malvern Instuments Ltd, Worcestershire, UK). Transmission electron micrographs (TEM) of the nanoparticles was assessed using JEOL JEM-1000 instrument (JEOL Ltd, Tokyo, Japan).

### Cell viability analysis

Cytotoxicity of HA-PEI/HA-PEG in SKOV-3TR or OVCAR8TR cells were assessed by MTT assay. In consideration of the consistency of the incubation time used in transfection, 5 × 10^3^ cells were seeded in 96-well plate in triplicate. On the following day, the cells were incubated with HA-PEI/HA-PEG for 6 h and then washed with fresh media to remove HA-PEI/HA-PEG. Subsequently, cells were cultured with fresh media for another 48 h and MTT assay was conducted. Briefly, 20 μL MTT (Sigma-Aldrich, St. Louis, MO) was added to each well and incubated for 4 h. After dissolving the resulting formazan products with acid-isopropanol, the absorbance was analyzed on a SpectraMax Microplate® Spectrophotometer (Molecular Devices LLC, Sunnyvale, CA) at a wavelength of 490 nm. Absorbance values were normalized by assigning the value of the cells in medium without nanoparticles to 100.

### Real-time PCR assay of *MDR1* expression

Real-time PCR was processed to identify the expression level of *MDR1* mRNA in SKOV-3TR or OVCAR8TR cells transfected with HA-PEI/HA-PEG/*MDR1* siRNA according to the manufacturer's protocol. In brief, RNA was extracted using Trizol Reagent® (Life Technologies) and reverse transcribed to cDNA by high capacity cDNA reverse transcription kits (Applied Biosystems, Foster City, CA). Equal amounts of cDNA were dispersed on the 96-well plate with the specific TaqMan® gene expression assay (Applied Biosystems) and TaqMan® gene expression master mix (Applied Biosystems). The amplifications were carried out on Applied Biosystems StepOnePlus™ System (Applied Biosystems). The Comparative CT method was used to analyze the relative *MDR1* mRNA expression level.

### Western blot assay

Western blot was conducted to determine expression of Pgp in SKOV-3TR cell line and mouse model. The cells were lysed with 1X RIPA lysis buffer (Upstate Biotechnology, Charlottesville, VA) supplemented with complete protease inhibitor cocktail tablets (Roche Applied Science, IN, USA). Protein concentrations were evaluated by the DC Protein Assay (Bio-Rad, Hercules, USA). Equal amounts of proteins were separated by NuPAGE® 4–12% Bis-Tris Gel (Life Technologies), transferred onto nitrocellulose membrane (Bio-Rad), and incubated with specific primary antibodies (dilution: 1:1000) at 4°C overnight. After extensive rinsing with TBST, the membranes were further probed with respective secondary antibodies (LI-COR Biosciences, Lincoln, NE) at 1:20000 dilutions. Finally, the membranes were scanned using Odyssey® CLx equipment (LI-COR Bioscences) to detect bands. The density of the bands was then quantified by Odyssey software 3.0 (LI-COR Bioscences).

### Immunofluoresecence assay

In order to further assess the expression levels of CD44 and Pgp in both ovarian cancer parental sensitive cells and multidrug drug resistant cells, immunofluorescence was performed as previously described^58^. Immunostainings were carried out using antibodies against CD44 and Pgp. After washing the cells, they were incubated with Alexa Fluor secondary antibodies. Specifically, the slides were stained with Alexa Fluor 594 (Red) conjugated for goat anti-mouse antibody (Life Technologies) for either CD44 or Pgp. Prior to microscopy, the nuclei were counter stained with 1 μg/mL Hoechst 33342 (Life Technologies) for 1 min.

### Tumor xenograft model and treatment

To determine the potential effects of the combination of HA-PEI/HA-PEG/*MDR1* siRNA and paclitaxel on tumor growth inhibition and elucidate underlying mechanisms to overcome MDR *in vivo*, SKOV-3TR cells (5 × 10^6^) were suspended in 1:1 mixture of RPMI 1640 and Matrigel (BD Biosciences, San Jose, CA), and then injected subcutaneously into Crl:SHO-*Prkdc*^SCID^*Hr*^hr^ nude female mice at 4-6 weeks age (Charles River Laboratories, Ann Arbor, MI). Animal procedures were performed according to an approved protocol by Massachusetts General Hospital Subcommittee on Research Animal Care (SRAC). The mice were randomized into 4 groups when the tumor volume reached approximately 200 mm^3^. The dosing schedule of treatment group was outlined in Fig 5A. The mice were administered with 0.5 mg/kg either *MDR1* siRNA loaded HA-PEI/HA-PEG, non-specific siRNA loaded HA-PEI/HA-PEG, *MDR1* siRNA alone or saline alone on day 1, 2 and 3. All groups were then followed with paclitaxel (20 mg/kg) treatment twice a week for two weeks beginning on the fifth day. The tumors volume (V_t_, mm^3^) was measured regularly twice a week and calculated using the formula (W^2^ × L)/2, where W represented width and L represented length. The relative tumor volume was calculated using the formula V_t_/V_0_, where V_0_ was tumor volume at time of treatment commencement. The mice were sacrificed and the tumors were collected on day 33. Part of tumor tissues were fixed in 4% buffered formalin and embedded in paraffin for immunohistochemistry staining and TUNEL apoptosis assay. Another part of tumor tissue was stored at −80°C for extraction of protein to examine the Pgp and CD44 expression levels.

### Immunohistochemistry

Immunohistochemistry was performed in an ovarian cancer tissue microarray (TMA)^54^ and in mice tumor tissues by using an Immunohistochemistry Protocol (Paraffin) and reagents from Cell Signaling Technology to detect the expression level of Pgp and CD44. Briefly, paraffin tissue sections from excised tumors were deparaffinized and hydrated through xylene and graded alcohol. Antigens were unmasked by heat treatment and endogenous peroxidase was inhibited by incubating with 3% H_2_O_2_ for 10 min. After blocking each section with blocking solution (Cell Signaling Technology) for 1 h at room temperature, they were incubated with primary antibody in a humidified chamber at 4°C overnight. Sections were then washed with TBST, and the signal were developed using SignalStain® Boost Detection Reagent (Cell Signaling Technology) and SignalStain® DAB (Cell Signaling Technology). Finally, the slides were mounted with VectaMount AQ (Vector Laboratories).

### TUNEL assay

To confirm the apoptosis level of tumor in mice, paraffin tissue sections of tumors were processed with the DeadEnd^TM^ Colorimetric TUNEL System (Promega, Madison, WI) according to the manufacturer's protocol.

### Statistics

The statistical analysis was done using Prism 5.0 software (Graph Pad Software Inc., San Diego, CA). The data were expressed as mean ± SEM and analyzed using two-tailed Student t-test or one-way ANOVA followed by Newman–Keuls post hoc test. Differences were considered statistically significant at *P* < 0.05.

## Author Contributions

Z.D., M.M.A., X.Y. and A.I. participated in the design of the study. X.Y., A.I. and A.S. acquired data. X.Y., A.I., F.J.H. and E.C. controlled analyzed and interpreted data. X.Y. and A.S. prepared the manuscript. Z.D., M.M.A., X.Y., A.I., A.S., F.J.H. and E.C. read, corrected and approved the final manuscript.

## Supplementary Material

Supplementary Information Supplementary Information
