# Supplementary material for: MDR1 siRNA loaded hyaluronic acid-based CD44 targeted nanoparticle systems circumvent paclitaxel resistance in ovarian cancer
Source: Sci Rep. 2015 Feb 17;5:8509. doi: 10.1038/srep08509 (PMC4330541; doi:10.1038/srep08509)
Supplement: Supplementary Information [file srep08509-s1.pdf]

MDR1 siRNA loaded hyaluronic acid-based CD44 targeted nanoparticle systems circumvent paclitaxel resistance in ovarian cancer

Xiaoqian Yang[1,2], Arun K. lyer[3,4], Amit Singh[3], Edwin Choy[1], Francis J. Hornicek[1], Mansoor M. Amiji[3], Zhenfeng Duan[1,*]

[1] Sarcoma Biology Laboratory, Center for Sarcoma and Connective Tissue Oncology, Massachusetts General Hospital and Harvard Medical School, Boston , MA 02114

[2] Department of Gynaecology and Obstetrics, The Third Affiliated Hospital of Zhengzhou University, Zhengzhou 450052, Henan Province, China

[3] Department of Pharmaceutical Sciences, School of Pharmacy, Northeastern University, Boston, MA 02114

[4] Current address: Department of Pharmaceutical Sciences, Eugene Applebaum College of Pharmacy and Health Sciences, Wayne State University, Detroit, MI 48201

Running title: *MDR1* siRNA delivery by CD44 targeted nanosystems

The authors disclose no potential conflicts of interest

* Corresponding author: Zhenfeng Duan, Sarcoma Biology Laboratory, Center for Sarcoma and Connective Tissue Oncology, Massachusetts General Hospital, 100 Blossom St, Jackson 1115, Boston, MA 02114, USA. Phone: 617-724-3144; Fax: 617-726-3883. E-mail address: zduan@mgh.harvard.edu

Supplementary Figure Legends:

Figure S1 | Expression of CD44 and Pgp in various ovarian cancer cell lines. Expression levels of CD44 and Pgp in ovarian cancer cell lines, including drug sensitive and resistant cell lines were determined by Western Blot.

Figure S2 | *MDR1* siRNA loaded HA-PEI/HA-PEG nanoparticles induced decreased Pgp expression in OVCAR8TR cells. OVCAR8TR cells were incubated with different agents as indicated. Pgp expression was significantly decreased in OVCAR8TR cells transfected with HA-PEI/HA-PEG/*MDR1* siRNA.

Figure S1

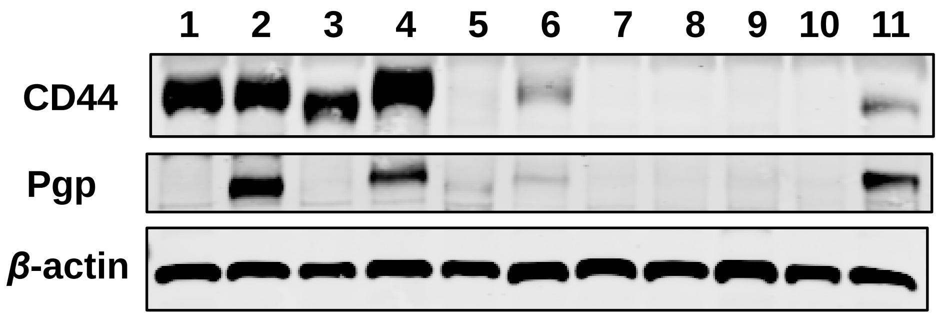

1: OVCAR8
2: OVCAR8TR
3: SKOV-3
4: SKOV-3TR
5: 2008
6: 2008cp
7: IGROV-1
8: IGROV-1cp
9: A2780
10: A2780cp
11: OVCAR5

Figure S2

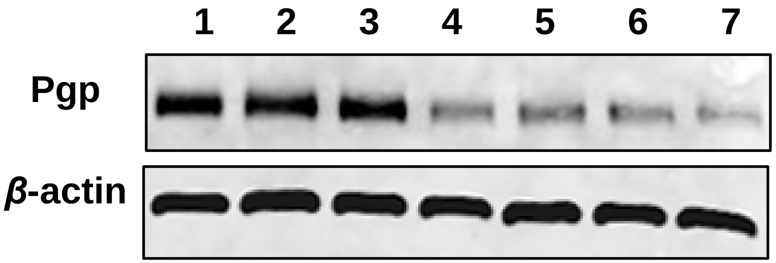

1: OVCAR8TR cell only
2: HA-PEI/HA-PEG/non-specific siRNA 45nM
3: *MDR1* siRNA alone 45nM
4: Lipofectamine /*MDR*1 45nM
5: HA-PEI/HA-PEG/*MDR1* siRNA 45nM
6: HA-PEI/HA-PEG/*MDR1* siRNA 90nM
7: HA-PEI/HA-PEG/*MDR1* siRNA 180nM
